# Leriche Syndrome Presenting with Multisystem Vaso-Occlusive Catastrophe

**DOI:** 10.5811/westjem.2015.4.25401

**Published:** 2015-06-24

**Authors:** C. Eric McCoy, Shaheena Patierno, Shahram Lotfipour

**Affiliations:** UC Irvine School of Medicine, Department of Emergency Medicine, Irvine, California

## INTRODUCTION

Leriche syndrome, also referred to as aortoiliac occlusive disease, has been described as a triad of claudication, impotence and decreased femoral pulses.^1^ The syndrome results from thrombotic aortoiliac occlusion and was first described by a French surgeon, Rene Leriche, in 1940.^1^–^2^ The disease most commonly occurs in men, and risk factors include hypertension, diabetes, hyperlipidemia, and smoking.^3^ Advanced diagnostic imaging techniques such as abdominal ultrasonography and computed tomography (CT) angiography assist the clinician in confirming the diagnosis. Treatment is primarily surgical and consists of aortoiliac endarterectomy and aortobifemoral bypass. Alternative procedures described are percutaneous transluminal angioplasty with stenting, and axillofemoral bypass.^4^–^5^

## CASE REPORT

A 56-year-old male was brought to the emergency department by paramedics for a syncopal episode and inability to move his lower extremities. He complained of abdominal pain and inability to move his legs beginning five hours prior to arrival. He awakened from a nap that afternoon and experienced numbness in both legs, which progressed to paralysis. At baseline, he was ambulatory without any history of weakness and was last ambulatory hours prior. He endorsed occasional pain in his legs when walking at baseline.

He also complained of abdominal pain with nausea and vomiting for two days. He had a history of alcohol use and reported dark-colored emesis and last bowel movement three days prior. On review of symptoms the patient denied any history of headache, dizziness, chest pain, back pain, trauma, fevers, or extremity weakness. He walked longer than a mile the day prior. History from the patient’s wife revealed that he had an episode of altered level of consciousness while on the couch and that finding combined with his abdominal pain and paralysis prompted her to call 911. The patient’s past medical history was significant for hypertension, peripheral arterial disease, and myocardial infarction 10 years prior. His past surgical history included “abdominal stents” and a left carotid stent. His social history was significant for a 25-pack/year history of smoking and daily alcohol. His medications included atenolol and ranitidine.

Physical exam showed an oral temperature of 36.7°C, blood pressure 107/65mmHg, heart rate 99 beats/minute and a respiratory rate of 30 breaths/min, with oxygen saturation on 15L non-rebreather mask of 94%. His weight was 72.5kg and he appeared older than his stated age. He was alert, cooperative and in moderate distress, primarily complaining of pain and cramping in his lower extremities and repeatedly asking staff to straighten out his legs although they were already lying straight and motionless on the gurney. His head exam was unremarkable with the exception of a dry oropharynx. Cervical spine, cardiac, and lung exams were unremarkable. His abdomen was firm and diffusely tender to palpation with generalized rebound and guarding. An irreducible left inguinal hernia was present. He had vomiting, and placement of a nasogastric tube revealed 1.5 liters of coffee-ground emesis. Lower extremities were thin, cool, and without any palpable or Dopplerable pulses in bilateral femoral, popliteal or pedal distribution. There was trace non-reproducible sensation to the mottled lower extremities, and no sensation distal to the ankles. Motor exam was significant for lower extremity paralysis.

Laboratory data consisted of sodium 121mEq/L (135–145), potassium 6.8mEq/L (3.3–4.8), chloride 89mEq/L (101–111), CO_2_ 18mEq/L (25–34), BUN 31mg/dL (8–26), creatinine 1.5mg/dL (0.5–1.3), and blood glucose 367mg/dL (70–115). White blood cell 19.1thous/mcL (4.0–10.5), hemoglobin 12.8g/dL (13.5–16.9), lipase 107U/L (22–51), hematocrit 38.8% (39.5–50.0), and platelets 165thous/mcL (150–400). There was a left shift in the neutrophils 16.2thous/mcL (85%) (2.0–8.1). Alkaline phosphate 88IU/L (26–110), AST 84IU/L (8–40), ALT 38IU/L (0.0–60), total bilirubin 1mg/dL (0.0–1.4), total protein 5.4g/dL (6.1–8.2), albumin 2.6g/dL (3.2–5.5). Lactate 7.2mmol/L (0.7–2.1). ABG showed pH of 7.25 (7.38–7.42), pCO_2_ 31.6mmHg (36–42), pO_2_ 123.6mmHg (80–104), bicarbonate 13.5mmol/L (21–27). PT was 17.3sec (9.5–12.3), PTT 45.9sec (24.1–35.1), and INR 1.62 (0.87–1.14). B-type natriuretic peptide 1,950pg/mL (<100). Troponin 2.75ng/mL (<0.03).

**Figure 1:**
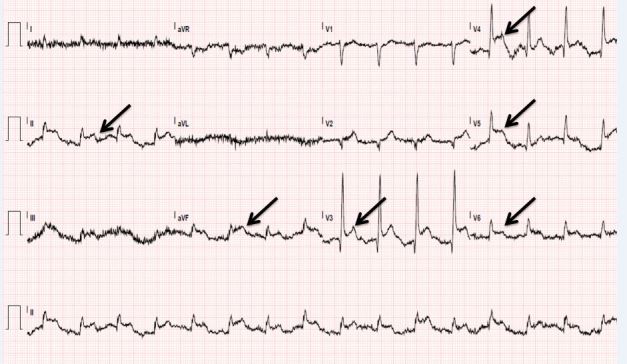
Electrocardiogram of patient with Leriche syndrome, demonstrating diffuse ST-segment elevation.

**Figure 2:**
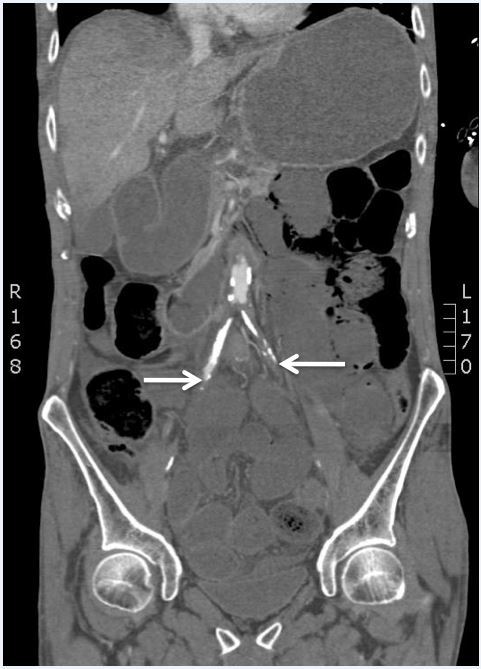
Coronal computed tomography demonstrating aortoiliac vascular occlusion.

Chest radiograph was unremarkable. Electrocardiogram (ECG) showed sinus rhythm at 95 beats/minute with ST elevation inferiorly, anteriorly and laterally (Figure 1). Bedside ultrasound to evaluate the abdominal aorta was limited. Vascular surgery was consulted prior to CT for concern of a vascular catastrophe. Cardiology was consulted for the patient’s ECG findings consistent with myocardial infarction. The patient went for a non-contrast head CT that was unremarkable and a CT angiogram of the chest, abdomen and pelvis, which was significant for the abdominal aorta with no contrast opacification 2.2cm superior to the bifurcation (Figure 2), high-grade stenosis of the right common iliac artery, complete occlusion of the left common iliac artery, stents in the celiac artery and superior mesenteric artery (SMA), evidence of occlusion of the proximal SMA and inferior mesenteric artery (IMA), hepatic, splenic, bilateral renal infarctions, left inguinal hernia, bowel obstruction, pneumatosis intestinalis with evidence of ischemic bowel, and aspiration in the right lower lung. General surgery was consulted.

### Patient’s Hospital Course

The cardiology service stated that the patient was not a candidate for cardiac catheterization and to start anticoagulation and low-dose aspirin if there was no contraindication or planned surgery. They also recommended thrombolytics for the diffuse thrombotic disease. The patient did have an echocardiography study that revealed an ejection fraction of 35% and multiple regional wall motion abnormalities. General surgery recommended comfort measures as he was not a surgical candidate. Vascular surgery commented that bypass would be futile and if patient survived, would be a candidate for extra-anatomical axillo-bifemoral bypass in the future. The patient was admitted to the medical intensive care unit for broad-spectrum antibiotics and a heparin drip and succumbed to his illness the following day.

## DISCUSSION

This patient presented with the complaint of sudden onset of lower extremity paralysis and was found to have severe vascular occlusive disease affecting the distal aorta, iliacs, SMA, and IMA, resulting in limb-threatening lower extremity infarction, hepatic, spleen, renal infarctions, and ischemic bowel. The patient had a STEMI, suggesting a completely occlusive thromboembolic event in the coronary(ies). He was also found to have a left inguinal hernia, bowel obstruction with pneumatosis intestinalis, and laboratory findings consistent with pancreatitis and acute kidney injury. These findings represent the end stage of the pathophysiologic basis of Leriche syndrome. To our knowledge, there is no published literature describing Leriche syndrome resulting in multisystem ischemia/infarction including the lower limbs, bowel, liver, spleen, kidneys, and myocardium.

Leriche syndrome was originally described as the syndrome of thrombotic obliteration of the aortic bifurcation.^1^ The typical presentation occurs in male patients with the clinical triad of intermittent claudication involving the low back, buttocks, hip or thigh, impotency and weak or absent femoral pulses.^5^–^6^ It mostly occurs in men in the third to sixth decades of life.^6^ The lower extremities can present with pallor, coldness and weakness. Our patient’s clinical presentation was compatible with the most severe end-stage clinical manifestations of this disease with complete aortoiliac occlusion and lower extremity paralysis. The patient did report a history that was consistent with intermittent claudication prior to the day of presentation and physical exam findings revealed absent femoral pulses. Although a history of impotence was not asked, the CT angiography findings of aortoiliac occlusion was consistent with an inability to maintain penile erection. Risk factors for this syndrome consist of hyperlipidemia, hypertension, diabetes mellitus and smoking.^3^ Our patient had a history of hypertension and smoking and laboratory findings with elevated blood glucose.

When this condition was first described in the 1940s, a clinician could make the provisional diagnosis of Leriche syndrome in a patient with the triad of claudication, impotence and decreased femoral pulses. Today, advanced diagnostic imaging techniques such as abdominal ultrasonography and CT angiography assist the clinician in diagnosis confirmation. Measurement of the ankle-brachial index aids in screening and is an indicator for peripheral arterial disease.

Aortoiliac occlusive disease mainly occurs in those patients with peripheral arterial disease. Atherosclerotic plaques cause symptoms by obstructing blood flow, and the unstable ones are prone to embolization to distal vessels. In advanced cases, although rare due to the chronic nature of the occlusive process, ischemia can occur. Thus, the underlying pathology of Leriche syndrome results from obstructing plaques due to atheromatous formation in the infrarenal aorta and iliac arteries.^6^–^7^

The syndrome begins at the distal aorta or common iliac arteries and progresses both proximally and distally over time.^8^ The same pathology that caused the classic findings of Leriche syndrome in this patient resulting in claudication, paralysis, and absent femoral pulses was present systemically as evidenced by infarction of the patient’s bowel, liver, spleen, kidneys, and heart.

The treatment for Leriche syndrome is primarily surgical and consists of aortoiliac endarterectomy and aortobifemoral bypass; alternative procedures described are percutaneous transluminal angioplasty with stenting, and axillofemoral bypass.^4^–^5^

The ideal yet rare surgical candidate for chronic aortoiliac thrombosis is the patient with negligible atherosclerotic involvement of the remainder of the vasculature.

## CONCLUSION

Leriche syndrome is a triad of claudication, impotence and decreased femoral pulses as a result of aortoiliac occlusion. The disease takes several decades to develop and if not recognized can lead to significant morbidity and mortality. This report is the first to our knowledge describing a patient with Leriche syndrome resulting in ischemia/infarction of the lower extremities, bowel, liver, spleen, kidney, and myocardium.
